# Photosynthesis by nonphotosynthetic microorganisms via semiconductor photocatalysis

**DOI:** 10.1002/mlf2.12156

**Published:** 2024-12-24

**Authors:** Bo Wang, Liang Shi, Anhuai Lu

**Affiliations:** ^1^ CAS Key Laboratory of Quantitative Engineering Biology, Center for Materials Synthetic Biology, Shenzhen Institute of Synthetic Biology Shenzhen Institutes of Advanced Technology, Chinese Academy of Sciences Shenzhen China; ^2^ Department of Biological Sciences and Technology, School of Environmental Studies China University of Geosciences Wuhan China; ^3^ Beijing Key Laboratory of Mineral Environmental Function, Key Laboratory of Orogenic Belts and Crustal Evolution, School of Earth and Space Sciences Peking University Beijing China

Photosynthesis has been the cornerstone of solar energy conversion on Earth for billions of years, crucial for sustaining the biosphere and maintaining the carbon and water cycles. However, with the rise of industrial civilization, natural photosynthesis alone has become insufficient to meet growing energy and product demands^1^, leading to extensive fossil fuel use, environmental pollution, and energy crises. In response, scientists have sought to enhance natural photosynthesis through genetic engineering and synthetic biology^2^, or to develop alternative solar conversion methods using advanced materials science^3^. Efforts to improve natural photosynthesis in plants and photosynthetic microorganisms have focused on optimizing light‐harvesting, electron transport, and carbon sequestration systems^2^. While photosynthetic microorganisms offer higher efficiency than plants^4^, their practical application is hindered by limited genetic tools, a narrow product spectrum, and slower growth compared to industrialized nonphotosynthetic microorganisms, which, despite their versatility, depend on organic or inorganic molecules for energy, leading to carbon costs and emissions issues^5^. In parallel, artificial solar‐to‐fuel/chemical conversion technologies^3^, particularly those using synthetic semiconductor materials, have gained prominence^6^. These materials have superior light‐harvesting capabilities and customizable band structures, leading to significant advances in photocatalysis and solar energy conversion efficiencies far exceeding those of natural photosynthesis^7^. However, the limited catalytic specificity of semiconductor photocatalysts restricts their product range to low‐value compounds, and the high cost, complex synthesis, and lack of environmental compatibility of most artificial semiconductors further underscore the significant challenges that remain before artificial photosynthesis can commercially yield more valuable products^8^.

## DISCOVERY OF SOLAR UTILIZATION BY NONPHOTOTROPHS

**Figure 1:**
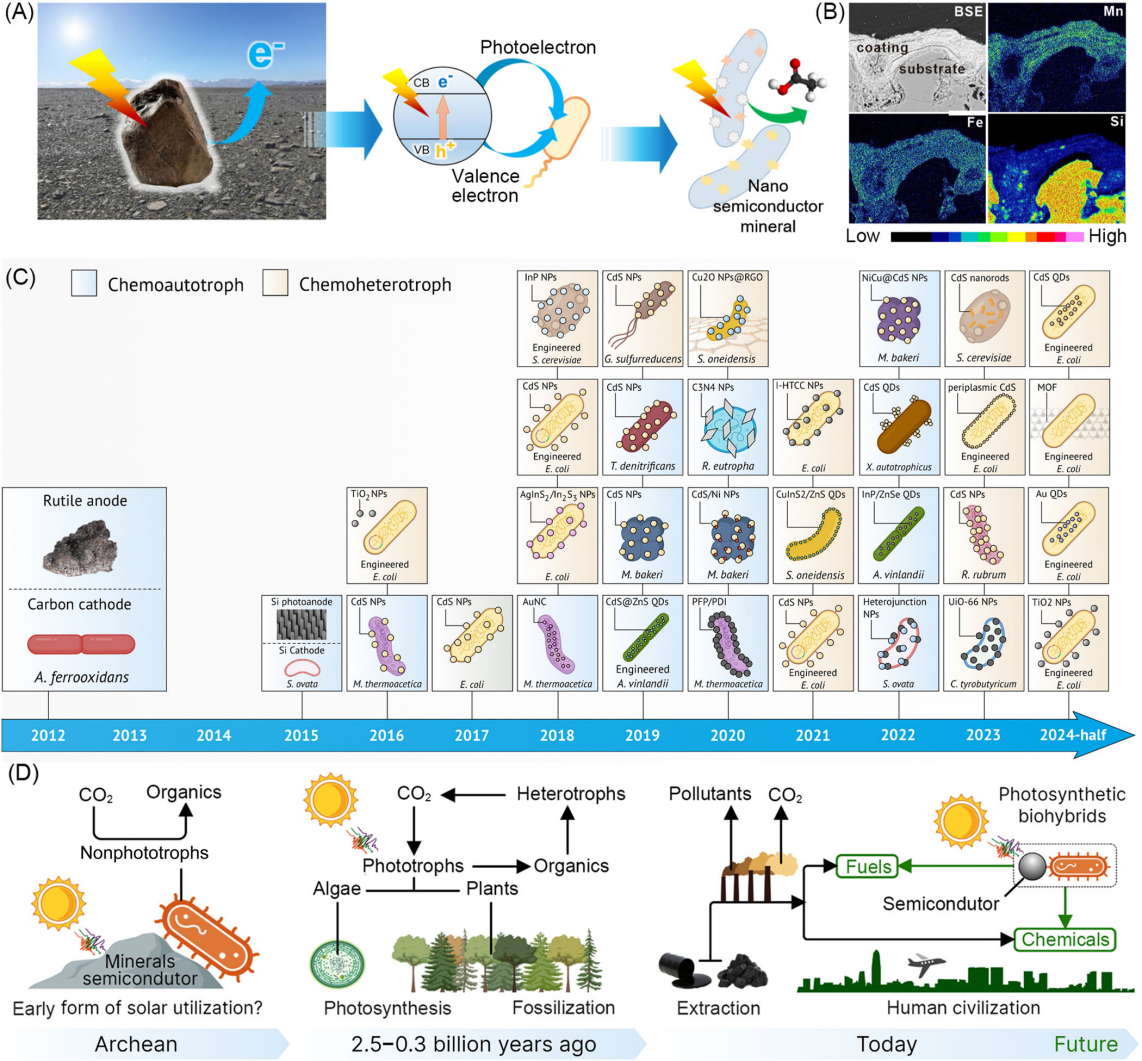
Exploring earth's mineral resources and developing semiartificial photosynthesis. (A) The abundant light‐harvesting minerals on the Earth's surface, possessing photoelectron conversion capabilities, can integrate with bacteria to facilitate light‐driven biosynthesis^10^. (B) Natural minerals rich in redox‐reactive elements^10^. (C) Timeline of the recent years' representative studies of the photosynthesis by nonphotosynthetic microorganisms. (D) Significance of studying the photosynthesis by nonphotosynthetic microorganisms.

In fact, there are numerous semiconductor materials in nature, specifically natural minerals rich in redox‐reactive elements such as iron, sulfur, and manganese^9^. These minerals can function as photocatalysts, harvesting solar energy for photoelectric conversion (Figure 1A,B)^10^. In 2012, we discovered that natural minerals with semiconductor properties can drive the iron cycle under light, thereby promoting the carbon sequestration and autotrophic growth of *Acidithiobacillus ferrooxidans* ^11^, a typical nonphotosynthetic bacterium. This marked the first report of nonphotosynthetic microorganisms utilizing photoelectrons from natural semiconductor minerals. Mineral–microbe interactions are widespread across various spatial and temporal scales, spanning from the early history of earth to the present^12^. Before our study, it was generally accepted that valence elements in minerals serve as substrates for redox reactions (acting as electron sinks or donors) during microbial metabolism. Minerals contain multivalence metal elements, such as Fe and Mn, which can act as electrical conductors to facilitate extracellular electron transfer^9^. Our finding revealed another form of solar energy utilization by microbes, likely present very early in nature: the harvesting and utilization of solar energy with the help of natural semiconductor minerals. Given that the evolutionary history of nonphototrophs is longer than that of phototrophs, it can be speculated that this natural phenomenon has existed for a considerable period of time.

Achieving photosynthesis in nonphotosynthetic microorganisms not only represents a landmark scientific breakthrough but also carries profound practical significance. First, it would help develop new platforms for solar energy harvesting and conversion, optimizing the use of solar energy to benefit humanity. Second, it could establish novel solar‐to‐fuel/chemical conversion pathways that are difficult to achieve in natural photosynthetic organisms, thus enabling the green biomanufacturing of numerous valuable products. Furthermore, this approach will pave the way toward the production of a diverse range of biochemicals in a carbon‐neutral manner. Since our pioneering study in 2012, more nonphotosynthetic microorganisms, including chemoautotrophs and chemoheterotrophs, have been verified to acquire energy from photoelectrons via biomineralized or chemically synthesized semiconductors (Figure 1C). This emerging research direction has become a hot topic and a key component of the burgeoning field of Semiconductor Synthetic Biology (Roadmap 2018)^13^. Recent reviews have introduced novel terms such as semiartificial photosynthesis and photosynthetic biohybrid systems to describe these advancements^14^.

## PHOTOSYNTHESIS BY CHEMOAUTOTROPHS

Chemoautotrophic microorganisms, which utilize energy from the oxidation of reduced inorganic chemicals for organic biosynthesis via CO_2_ fixation, inherently possess pathways to directly or indirectly use extracellular electrons^15^. In a photoelectrochemical chamber constructed by Liu et al. in 2015, a TiO_2_ nanowire array on the anodes harvested light energy, producing photoelectrons and H^+^ from H_2_O. These anodes were connected to silicon (Si) nanowire cathodes covered with a biofilm of *Sporomusa ovata*, which successfully used the photoelectrons and H^+^ from the anodes to convert CO_2_ into acetate^16^. In 2016, a CdS–*Moorella thermoacetica* biohybrid was created by incubating the bacterium in the presence of Cd^2+^ and sulfur‐containing cysteine^17^. Semiconductive CdS nanoparticles precipitated on the cell surface, harvesting light energy to produce photoelectrons extracellularly. These photoelectrons were then directly transferred into bacterial cells via various pathways and utilized for the reduction of CO_2_ to acetate. In recent years, many other acetogens, methanogens, and electroactive species have also been investigated in biohybrid construction^14^. Due to the lack of genetic tools, most chemicals derived from these biohybrids are natural products with limited value. Further product upgrading needs to be achieved by combining these biohybrids with other heterotrophic microorganisms, such as *Escherichia coli* and *Rhodopseudomonas palustris*, to form microbial consortia^16^, ^18^, or by feeding the products of chemoautotrophs to a secondary microbial culture for metabolic conversion, as implemented in photoelectrocatalytic–biocatalytic flow systems^19^.

## PHOTOSYNTHESIS BY CHEMOHETEROTROPHS

Reports of chemoheterotrophic microorganism‐based biohybrids have increased rapidly since 2017. Chemoheterotrophic microorganisms, particularly model strains, offer several advantages, including well‐developed genetic tools, high growth rates^20^, and good stability under industrial conditions^5^. For example, the widely used *Saccharomyces cerevisiae* in the biomanufacturing industry was combined with InP semiconductor nanoparticles using a modular assembly strategy. The resulting biohybrid achieved solar‐driven shikimate production with an efficiency of 1.58%^21^. Additionally, *S. cerevisiae* was hybridized with intracellular CdS nanodots, achieving high‐efficiency solar‐driven H_2_ production^22^. The prokaryotic industrial workhorse *E. coli* was also used to construct biohybrid systems for generating a variety of target products driven by light^20^, ^23^. However, unlike chemoautotrophs, which contain many electroactive membrane proteins (such as OmcA, MtrA/B/C, CymA, OmcB, etc.), the electron transfer mechanism at the biotic–abiotic interface of chemoheterotrophs is quite obscure. Based on current understanding, it is very difficult to rationally construct a chemoheterotrophic microorganism‐based biohybrid system for efficient solar‐to‐fuel/chemical energy conversion. Moreover, the lack of native CO_2_‐assimilation pathways makes chemoheterotrophs less competitive in next‐generation biomanufacturing applications^5^. Although recent synthetic biology studies have successfully integrated CO_2_‐assimilation modules into *E. coli* and yeast cells^24^, more efforts are still needed to match and adapt the different exogenous modules to these chemoheterotrophic chassis cells.

## CHALLENGES

Despite recent advances, the field of photosynthesis with nonphotosynthetic microorganisms is still in its infancy. Scaling up these systems to be comparable to natural photosynthesis in a sustainable manner and transitioning this promising research area from the lab to real‐world applications require significant efforts to reduce material costs and improve overall solar conversion efficiency^20^, ^25^. In essence, the goal is to maximize solar energy benefits with minimal material input into the microbial system. If the solar energy benefits far exceed the additional material costs, biohybrid systems could become economically viable. In terms of inexpensive and readily available semiconductor materials, light‐harvesting minerals are widespread on earth's surface. In 2019, we discovered that thin layers of Fe‐ and Mn‐bearing minerals on rocks in the Gobi Desert, karst terrains, and soil particles in China functioned as excellent semiconductors capable of generating photoelectrons using sunlight^10^. The band gap of Fe/Mn‐bearing minerals is 1.77–2.57 eV, corresponding to a photosensitive wavelength range of 482–700 nm, effectively covering the visible light spectrum of solar radiation. Inorganic substances like H_2_O and transition‐metal ions, along with organics such as humic acid, all serve as electron donors for these light‐harvesting minerals. It was estimated that up to 2.23 × 10^16^ photoelectrons could be produced per second from 1 m^2^ of the thin layer of minerals^10^. The widespread availability of light‐harvesting minerals presents an abundant and cost‐effective resource for developing biohybrid systems geared toward solar‐to‐chemical biosynthesis of organic compounds. In addition, natural minerals, compared to synthetic semiconductors, offer a relatively straightforward preparation process, with rich doping possibilities and intrinsic vacancies that enhance light absorption and efficient charge separation. However, it is crucial to recognize the inherent limitations of natural minerals, such as their variability and lack of uniformity, which can hinder their practical application. Overcoming these challenges will necessitate the development of innovative, cost‐effective solutions. Enhancing energy transfer efficiency at the biotic–abiotic interface is crucial for overall solar conversion efficiency. However, due to current limitations in bioelectrochemistry and spectroscopy technologies, it remains challenging to accurately and in real time reveal the electron transfer mechanisms at the material–cell interface. Consequently, most reported biohybrid systems have been constructed using top‐down strategies with limited rationality. Addressing this challenge requires interdisciplinary collaboration among scientists from various fields. Broadening of the spectral range that can be captured by solar‐harvesting materials, enhancing materials' resistance tophotocorrosion and reducing light‐induced ROS generation, maximizing the power output of lighting devices and increasing microbes' tolerance to intense light irradiation, can be adopted to improve solar conversion efficiency. Furthermore, enhancing nanomaterial–cell adhesion, strengthening the affinities and specificities between target enzymes and nanomaterials (e.g., intracellular quantum dots) or energy carriers (e.g., methyl viologen and neutral red), and unveiling the unclear pathways of energy flow are feasible measures to optimize energy transfer at the biotic–abiotic interface^25^. Advanced synthetic biology techniques, including chassis cell design, metabolic engineering, and directed evolution, are expected to play pivotal roles in the future development of biohybrid systems^25^. Other challenges facing the future development of biohybrid systems include the limited availability of engineering tools to optimize the chemical productivity for candidate microbial strain, the robustness of microbial cells to survive the working lifespan under inhospitable conditions, and the accidental leaking of genetically modified microorganisms and potentially biohazard nanomaterials without the understanding of their environmental implications. We believe that a rationally constructed biohybrid system with highly efficient solar‐to‐fuel/chemical energy conversion, along with a modular assembled material–cell interface enabling the recyclable use of materials, would further reduce system costs and facilitate practical applications.

By drawing inspiration from nature and applying it to human society, we aim to explore the potential of studying photosynthesis in non‐photosynthetic microorganisms using semiconductors. This approach not only enhances our understanding of ancient microbial utilization of solar energy (Figure 1D) but also lays the foundation for harnessing solar energy effectively, developing efficient and environmentally friendly synthetic pathways for diverse products, and ultimately contributing to the sustainable development of human society.
